# Gastric sleeve surgery in hemodialysis: A case report

**DOI:** 10.1016/j.ijscr.2019.02.041

**Published:** 2019-03-05

**Authors:** Kateir Mariel Contreras Villamizar, Diana Carolina Afanador Rubio, Camilo Alberto González González, Paola Karina García Padilla, Martha Patricia Rodríguez Sánchez

**Affiliations:** Nephrology Unit, Internal Medicine Department, Hospital Universitario San Ignacio, Medical School, Pontificia Universidad Javeriana, Colombia

**Keywords:** CKD, chronic kidney disease, BMI, body mass index, ESRD, end-stage renal disease, BIA, bioelectrical impedance, DW, dry weight, RYGB, Roux-en-Y gastric bypass, LSG, laparoscopic sleeve gastrectomy, LAGB, laparoscopic adjustable gastric banding, DDP, dialysis-dependent patients, NDP, non-dialysis dependent patients, PMS, post-surgical morbidity, Bariatric surgery, Renal dialysis, Electric impedance, Obesity

## Abstract

**Introduction:**

Morbid obesity in chronic kidney disease patients on hemodialysis limits access to renal transplantation. We report here a case of a surgical procedure for weight reduction in a hemodialysis patient and adjustment of dry weight through bioelectrical impedance.

**Case presentation:**

A 44-year-old male with CKD on hemodialysis for 26 years. After 3 years on dialysis, he underwent a cadaveric kidney transplant. However, after 8 years of transplant, he loses the kidney graft and returns to dialysis treatment. The patient’s BMI increased to 42 kg/m^2^ and he had difficult-to-control hypertension and severe sleep apnea. Behavioral, nutritional and pharmacologic measures were not sufficient to achieve an adequate weight control. Thus, a surgical procedure for weight reduction was considered. The patient underwent a laparoscopic gastric sleeve without any complications. Dry weight was adjusted through bioelectrical impedance before each hemodialysis session. The patient did not display hypotension, cramps, or fluid overload. After a 30 kg weight loss, the patient’s BMI was 28.3 kg/m^2^, allowing registration on the kidney transplant waitlist.

**Discussion:**

Obesity in CKD restricts access to kidney transplant waitlist. Bariatric surgery has proven to be safe and effective for sustained weight loss and it seems that the fact that a patient is dialysis dependent does not independently increase post-operatory complications.

**Conclusion:**

Surgical procedures for weight reduction in dialysis patients does not independently increase the risk for adverse outcomes after bariatric surgery. The estimation of DW through BIA is an effective method for avoiding complications generated by excessive or deficient ultrafiltration.

## Highlights

• Obese patients with end-stage renal disease on hemodialysis, decreases the access to kidney transplantation.
• Bariatric surgery has proven to be safe and effective for weight loss.
• Dialysis-dependency does not independently increase the risk for adverse outcomes after bariatric surgery.
• The estimation of DW through BIA is an effective method for avoiding complications generated by excessive or deficient ultrafiltration.

## 1 Introduction

Obesity is a main issue for healthcare systems worldwide [1]. In 1998, the World Health Organization established criteria for defining overweight and obesity as follows: Overweight: body mass index (BMI) above 25 kg/m^2^, Class 1 obesity: BMI 30–34.9 kg/m^2^, Class 2 obesity: BMI 35–39.9 kg/m^2^ and Class 3 obesity: BMI above 40 kg/m^2^ [2]. Having a higher BMI increases cardiovascular risk and reduces life expectancy, while becoming an independent risk factor for development and progression of chronic kidney disease (CKD) [2,3].

Obese patients with end-stage renal disease (ESRD) on hemodialysis seem to have greater survival rates, at least in the short term [2]. However, the access to kidney transplantation decreases. This can be explained by frequent graft loss, post-operatory complications, higher risk for new-onset diabetes after the procedure, and technical factors that increase surgical complexity in these patients. Thus, most transplant centers have established BMI-related criteria for defining inclusion in waitlist [4,5].

Bariatric surgery has proven to be safe and effective for sustained weight loss and adequate control of obesity-related underlying diseases [6]. Therefore, the National Institutes of Health have proposed specific indications for bariatric surgery [7] (Table 1).

**Table 1:** Indications for Bariatric Surgery.

BMI ≥ 40 kg/m^2^ or
BMI > 35 kg/m^2^ with at least 1 co-existing disease: • Systemic hypertension • Diabetes mellitus • Sleep apnea • Arthropathy • Coronary artery disease
Having made at least 1 attempt in a structured weight loss program.

Therefore, the decision to perform surgery in obese dialysis-dependent patients should be individualized.

Herein, we present the case of a morbidly obese patient on hemodialysis who underwent bariatric surgery with favorable results and was then registered on the kidney transplant waitlist.

This work was reported according to the SCARE guidelines [8].

## 2 Case presentation

A 44-year-old male patient was admitted to our institution with a past medical history remarkable for CKD as a result of congenital left renal hypoplasia and adaptive focal segmental glomerulosclerosis due to long-term obesity. The patient started peritoneal dialysis at the age of 26. After 3 years, he underwent a cadaveric kidney transplant. During the postoperatory period, slow graft function was seen, which recovered satisfactorily over time. Immunosuppressive therapy was initiated with cyclosporine, mycophenolate mophetil and prednisolone. Nevertheless, multiple celular rejection episodes lead to chronic graft glomerulopathy and hemodialysis requirement after 8 years. At the time, the patient’s BMI was 30 kg/m^2^. Progressively, weight gain increased BMI to 42 kg/m^2^ with difficult-to-control hypertension and severe sleep apnea. At that point, the patient’s waist circumference was 120 cm and fasting glucose was 100 mg/dL. As for the lipid panel, total cholesterol was 145.8 mg/dL, high-density cholesterol was 33 mg/dL and triglycerides were 117.1 mg/dL. Uric acid was 7.9 mg/dL and albumin was 3.9 g/L. Regarding dialysis adequacy parameters: single-pool Kt/V was 1.47, body fat measured through bioelectrical impedance (BIA) was 45%, phosphorus was difficult to control, and oscillated between 5 and 8 mg/dL.

Behavioral, nutritional and pharmacologic measures were not sufficient for an adequate weight control, thus in order to allow access to a second kidney transplant, the patient’s case was brought to consideration by a multidisciplinary board where a surgical approach was decided. A gastric sleeve was performed. There were no early or late post-operatory complications after a 12-month follow-up period.

On the initial dialysis sessions after surgery, due to rapid weight loss, the patient displayed cramps, dehydration and hypotension, which required several adjustments of dry weight (DW) by BIA, almost daily at first and weekly thereafter in order to better define the optimal ultrafiltration rate and symptom resolution.

The patient achieved a BMI of 28.6 kg/m^2^, adequate blood pressure control and reduced severity of sleep apnea. He then met all required criteria, and was registered in the kidney transplant waitlist.

## 3 Conclusions

Bariatric surgery has been steadily becoming safer due to increasing surgical experience, minimally invasive techniques and better post-operatory care. Gastric sleeve is an effective technique for weight reduction. Dialysis-dependency does not independently increase the risk for adverse outcomes after bariatric surgery. Nonetheless, comorbidity in this group of patients may increase surgical risk. The estimation of DW through BIA is an effective method for avoiding complications generated by excessive or deficient ultrafiltration. Our patient had an adequate clinical progression after undergoing bariatric surgery. There were no complications associated to the surgical procedure and the patient is currently a good candidate for future kidney transplantation.

## 4 Discussion

Obesity is an issue for kidney transplantation programs, since morbid obesity in ESRD is a growing epidemic [7,9], and one of the main causes for temporary inactivation from waitlists, thus most transplant centers restrict access to waitlists based on BMI, with cut-off values around 35 kg/m^2^ [4,10]

Achieving a negative caloric balance by adjusting intake and caloric expenditure is the cornerstone of weight loss. However, those who achieve an adequate weight loss are at high risk for regaining the lost weight during the early post-transplant period [7,11].

On the other hand, bariatric surgery has proven to be safe and effective for sustainable weight loss and suitable control of underlying diseases [6]. Surgical procedures for weight loss can be classified into two main categories: restrictive and malabsorptive (leading to lower caloric intake and absorption). These procedures include Roux-en-Y gastric bypass (RYGB), laparoscopic sleeve gastrectomy (LSG) and the laparoscopic adjustable gastric banding (LAGB) [12].

RYGB is considered the surgical “Gold-standard” for the treatment of obesity. Nonetheless, LSG is an effective procedure for weight control, with reported weight loss ranging between 51 and 70%; it is technically easier to accomplish, and has a lower risk of malnutrition, since this is a restrictive-type procedure. The latter should also be taken into account, since procedures leading to malabsorption, such as the RYGB may affect absorption of immunosuppressive therapy and thus generate complications once the transplant has taken place [12,13].

However, even if good results have been reported in the general population, the risks of performing this surgical procedure in CKD patients have not been established [9]. A study performed by Turgeon et al, analyzed 27,736 patients who underwent bariatric surgery between 2006 and 2008; 34 of these subjects were on dialysis. From those with CKD without dialysis requirement, 75% had a normal glomerular filtration rate, 18% had Stage-2-CKD, 6.25% had Stage-3-CKD, 0.34% had Stage-4-CKD and 0.33% had ESRD. The results showed that CKD is directly associated with the complication rate (an OR of 1.3 for every stage of progression of CKD was seen). Nevertheless, the absolute risk was below 10% [14].

Further studies have evaluated the safety and efficacy of LSG in patients with renal dysfunction. A retrospective review of patients with morbid obesity on the waiting list for kidney and liver transplant documented a 50% loss of their baseline weight in 12 months. This study reported 6 post-operatory complications: two superficial incisional surgical site infections, one wound dehiscence, one hemorrhage which required transfusion of red blood cells, one case of delirium and one case of acute kidney injury. After surgery, all patients were registered on the kidney transplant waitlist [13].

Furthermore, the biggest study conducted in order to analyze results in bariatric surgery patients with morbid obesity and CKD on dialysis was performed by Andalib et al. Data was extracted from the American College of Surgeons National Surgical Quality Improvement Program database. This study included information regarding patients with morbid obesity that underwent LAGB, LSA or RYGB. 234 dialysis-dependent patients (DDP) and 113,911 non-dialysis dependent patients (NDP) were included for analysis. The findings revealed a higher 30-day post-surgical morbidity (PSM) rate in DDP than in NDP (5.98 vs.2.31%; p < 0.001). Nonetheless, these patients were older (mean age 47.26 ± 10.38 in DDP vs. 44.72 ± 11.6 in NDP p < 0.001), and had a higher prevalence of hypertension and coronary artery disease than their counterparts. The multivariate analysis revealed that being dialysis-dependent was not an independent prognostic factor for 30-day PSM (OR 1.59; 95% CI 0.64–3.97). It seems that being dialysis-dependent does not independently increase 30-day post-operatory complications, comorbidity does [9].

DW is defined as the lowest tolerated post-dialysis weight once fluid overload and abnormal blood pressure have been excluded [15]. Currently, the adjustment of DW relies on clinical judgment, which may lead to an inexact determination and over or underestimation of this variable. The latter increases the risk for hyper/hypotension, left ventricle hypertrophy, arrhythmias, vascular access thrombosis and poor tolerance to treatment [16]. Therefore, the use of BIA techniques is a non-invasive and highly reproducible approach to determine fluid volumes across body compartments. DW can be estimated by measuring the extracellular fluid content (EFC) and total body water (TBW), and then performing an EFC/TBW quotient. Multiple cohort studies and randomized trials have established that weight loss based on serial estimations of EFC through BIA improves blood pressure control as well as echocardiographic determinants of left ventricular mass. Furthermore, a euvolemic status is associated with better long-term survival for DDP. The use of BIA in our case allowed precise adjustment of DW and avoidance of related complications [17].

## Conflict of interest

We have no competing interests.

## Funding sources

This research did not receive any specific grant from funding agencies in the public, commercial, or not-for-profit sectors.

## Ethical approval

All necessary measures were taken in order to keep relevant information confidential. The patient signed an informed consent, and followed protocols regarding publication of patient-related information as outlined by the Hospital Universitario. The ethics committee of our institution approved the conduction of the present study.

## Consent

Written informed consent was obtained from the patient for publication of this case report.

## Author contribution

Kateir Mariel Contreras and Diana Carolina Afanador contributed to study design, data collection, data interpretation, and writing the paper.

Camilo Alberto Gonzalez, Paola Karina Garcia y Martha Patricia Rodriguez contributed to study design, data collection and data interpretation

## Registration of research studies

This manuscript is not a human study, but a case report.

## Guarantor

Kateir Mariel Contreras Villamizar.

## Provenance and peer review

Not commissioned, externally peer reviewed.
